# Effect of the practice environment of nurses on job outcomes and safety climate*

**DOI:** 10.1590/1518-8345.2633.3056

**Published:** 2018-10-25

**Authors:** Gisele Hespanhol Dorigan, Edinêis de Brito Guirardello

**Affiliations:** 1 Fundação Hermínio Ometto, Uniararas, Araras, SP, Brazil.; 2 Universidade Estadual de Campinas, Faculdade de Enfermagem, Campinas, SP, Brazil.

**Keywords:** Health Facility Environment, Patient Safety, Job Satisfaction, Quality of Health Care, Nursing, Health Management

## Abstract

**Objective:**

to propose and analyze a theoretical model to measure the effect of nurses’ perceptions about the practice environment on safety climate, job satisfaction, intention to stay employed and in the profession, and burnout syndrome.

**Method:**

correlational study with probabilistic sample of 465 nurses. In the theoretical model, the dimensions of the nursing practice environment were considered as independent variables and job satisfaction, safety climate, intention to stay employed and in the profession, and burnout were considered the outcome variables. Structural Equation Modeling was the method used in the analysis.

**Results:**

small adjustments were made in the model and the dimensions of practice environment predicted job satisfaction (R^2^ = 43%), safety climate (R^2^ = 42%) and burnout (R^2^ = 36%), as well as the intention to stay in the job (R^2^ = 22%) and in the profession (R^2^ = 17%).

**Conclusion:**

the practice environment showed a strong impact on job satisfaction, safety climate and burnout, with a moderate impact on the intention to stay in the institution and in the profession. The findings can be used to manage care in health institutions, focusing on promoting nurse retention and improving the safety climate.

## Introduction

Planning human resources in health and aspects related to patient safety assessment are priorities for health organizations. When it comes to the adequate management of human resources, there is a concern with nursing professionals with respect to the fact that, in addition to providing uninterrupted patient care and making up the majority of health professionals, they are in a position of strategic insertion at the most different levels of attention and assistance scenarios. It is precisely because they are in this position that the assessment of the nurses’ perception of the organizational characteristics of the work environment can provide information to improve the quality of care and safety climate^1^.

A broad range of studies presents the positive results of the perception of the nursing practice environment on variables called job “outcomes”. Such results include increased job satisfaction, decreased intention to leave the profession and reduced levels of *burnout* ^2^ ^-^ ^5^; among patients, there is a reduction in mortality in institutions^6^ ^-^ ^7^.

The environments to which nurses are exposed are not always favorable to their practice. This situation highlights the importance of studies evaluating and reflecting on organizational safety culture. Researches that relate the perception of the practice environment to the evaluation of safety climate by nurses have been recommended due to the still incipient number of studies, especially national studies, on the theme^1^ ^,^ ^8^.

Considering previous research as a reference, a theoretical model based on the following research question was proposed: What are the effects of the nursing practice environment on job satisfaction, safety climate, burnout, and the intention to stay in the job and in the profession?

This study aims to propose and analyze a theoretical model that measures the effect of nurses’ perceptions about the practice environment on safety climate, job satisfaction, intention to stay employed and in the profession, and burnout syndrome.

## Method

This is a correlational study with quantitative approach. For the creation of a theoretical model, we considered the construct of nursing practice environment as an independent variable of second order composed of three constructs (autonomy, control over the environment and nurse-physicians relationships); the other constructs were perceived as dependent variables: safety climate, job satisfaction, intention to stay (in the current job in the next year and in the nursing profession) and burnout.

Previous studies^(2-4, 8-9)^ supported the raising of the hypothesis regarding the positive perception of nurses of the practice environment: 1) low levels of burnout; 2) greater intention to stay in the current job in the next year; 3) greater intention to stay in the profession; 4) greater job satisfaction and 5) more positive perception of safety climate.

The statistical method of Structural Equation Modeling (SEM) was used to estimate the minimum sample size calculated using the free *software* G*Power 3.1^10^. The second order construct (nursing practice environment) was considered as the independent variable of the model, an average effect size of 0.15 and power of test of 0.80 were adopted, obtaining 55 cases. It is recommended to use the triple of this value^10^; thus, a minimum sample of 165 nurses was estimated according to this parameter.

To calculate the sample size, the population of 104,397 nurses registered at the Regional Nursing Council of São Paulo (COREN-SP), Brazil, was used. Assuming a sample error of 3% and a significance level of 5%, the sample size calculated was 1057 subjects. Nurses were selected through random simple probabilistic sampling and the draw resulted in 1516 subjects; the final sample was composed of 465 subjects.

We included nurses with active professional registration in COREN-SP and who reported developing activities of direct assistance to patients, in a position of supervision in health institutions and having a time of experience equal to or greater than six months in the institution in which they performed their professional activities. Nurses who reported to be exclusively engaged in teaching, management or supervision activities at the institution were excluded, as they did not always carry out direct care activities exclusively with patients. Nurses who reported absence from work due to medical leave for any reason or any other type of leave in the period of data collection were also excluded.

Data collection took place from December 2014 to June 2015 through online means, through a partnership with the COREN-SP. For the selection of the participants, electronic invitations were sent by a computer professional through randomization registration numbers of these professionals.

The perception of nurses on the practice environment was evaluated through the Nursing Work Index Revised (NWI-R)^11^, composed of four subscales: autonomy (5 items), control over the environment (7 items), nurse-physicians relationships (3 items) and organizational support (10 items). This last subscale, however, was not used because it contemplated the same items as the previous ones, which could compromise the measurement and the quality of the model to be tested.

A four-point response Likert-type scale was used where the lower the value, the more positive is the perception of the practice environment. Values equal to or less than 2.5 points are understood to indicate favorable environments. Cronbach’s alpha values in the present study were 0.80 for the subscales of autonomy and control over the environment and 0.88 for the subscale nursing-physician relationships.

Safety climate and job satisfaction were measured through the subscales of job satisfaction (5 items) and safety climate (7 items) of the Safety Attitudes Questionnaire - Short Form 2006 (SAQ)^12^. The questionnaire had a five-point response Likert-type scale, also offering the possible answer “not applicable” for which no score is attributed. Scores of the subscales are obtained by the mean of the scores of the items answered and it is considered that scores above 75 indicate satisfaction at the job and a positive evaluation of safety climate. In this study, the Cronbach’s alpha coefficient was 0.85 for the job satisfaction subscale and 0.77 for the safety climate.

To evaluate the level of burnout, the Brazilian version of the Maslach Burnout Inventory (MBI) was used, including the subscales emotional exhaustion (9 items), depersonalization (5 items) and decreased personal accomplishment (8 items). The response scale has five points and the scores were analyzed through tertiary intervals for categorization of burnout levels into high, medium and low. Cronbach’s alpha coefficient values in this study resulted in 0.92 for emotional exhaustion, 0.68 for depersonalization and 0.81 for decreased personal accomplishment.

The data were organized in a spreadsheet and subjected to descriptive analysis with the SAS^®^ *software* version 9.2. For the analysis of the theoretical model using the Partial Least Squares Path Modeling (PLS-PM) approach, the SmartPLS 3 software was chosen. Firstly, the measurement model was evaluated using the Average Variance Extracted (AVE), obtained to each of the model constructs. AVE values above 0.50 indicate adequate convergent validity and, if they these values are not present, the items with the lowest factor loads are excluded one by one^10^ until values greater than 0.50 are obtained.

Composite reliability coefficients (CR) and the Cronbach’s alpha values were then evaluated, considering values greater than 0.70. Due to the greater sensitivity of the Cronbach’s alpha coefficient to the number of subscale items, it is recommended to use the PLS-PM analysis method to evaluate the internal consistency by means of the composite reliability coefficient which considers the factorial loads of the variables and is interpreted in the same way as Cronbach’s alpha^10^.

Cross loading values were observed in order to evaluate the discriminant validity; the items should show higher factorial load values in the construct they represent. The adherence to the criterion was assessed by the square root values of AVE that should be higher than the values of the correlations between the constructs^10^.

After checking the convergent and discriminant validity of the measurement model, the structural model analysis was started. The values and significance of path coefficients (Г) and the quality index of fitness of the tested model were analyzed, namely, the Pearson’s coefficient of determination (R^2^), the relevance or predictive validity (Q^2^), and the size of the effect (f^2^) for the outcome variables^10^. The significance of path coefficients was evaluated by the Bootstrapping method, considering the value of 5000 re-sampling. After adjusting the model, the interpretation of path coefficients considered the theoretical reference, through which the hypotheses were tested. As for the R^2^ values, it was considered that values equal or higher than 0.02 indicate a small effect, equal or higher than 0.13 indicate a medium effect; and equal or higher than 0.26, a large effect^10^.

As for relevance values or predictive validity, values above zero were considered adequate^10^. The contribution of the variables to the adjustment of the final model was evaluated by means of f^2^ values for the variables, categorized as small, medium and large, respectively: 0.02, 0.15 and 0.35^10^.

This research was approved by the Research Ethics Committee (CAAE 2014/ 30822314.9.0000.5404). A significance level of 5% was adopted in all statistical tests.

## Results

The sample consisted of 465 nurses, mostly females (84.09%), with mean age of 35.75 years (SD = 8.79), married (39.78%), without children (52.26 %) and with more than four years of experience in the institution (46.45%). The weekly average workload was 42.78 hours (SD = 13.19) and the majority had no other employment bond (78.28%). As to the area or unit of work, 36.98% of the nurses worked in medical-surgical clinic units; 16.14% in adult, pediatric and neonatal intensive care units; 23.01% in primary health care; 18.71% in outpatient units and psychiatric units; and 5.16% in private clinics or prison units. The analysis also indicated a mean value of 6.66 (SD = 3.39) for the intention to stay in the institution and 7.53 (SD = 3.11) for the intention to stay in the profession.

In relation to the practice environment, the mean autonomy was 2.29 (SD = 0.70), the mean for control over the environment was 2.52 (SD = 0.67) and for nursing-physician relationships was 2.22 (SD = 0.79). Job satisfaction presented a mean of 68.83 (SD = 23.69) and safety climate, a mean of 63.42 (SD = 19.50). The mean score of the MBI emotional exhaustion subscale was 25.55 (SD = 7.35), of depersonalization was 10.27 (SD = 3.58) and of decreased personal accomplishment was 30.15 (SD = 4.55).

In the evaluation of the initial model, AVE values ​​lower than 0.50 were obtained in four items excluded from the model to assure the convergent validity, such as: item 15 of the subscale control over the environment (“The designation of patients promotes continuity of care, that is, the same nurse takes care of the same patients on consecutive days”); item 11 of the subscale safety climate (“In this area, it is difficult to talk about errors”); item 15 of the subscale depersonalization (“I really care about what happens to some of my patients”) and item 4 of the subscale decreased personal accomplishment (“I can easily understand what my patients feel about the things that happen in the day-to-day”). The composite reliability values for all constructs of the proposed model ranged from 0.81 to 0.93 and Cronbach’s alpha coefficient values ranged from 0.68 to 0.92 (Table 1).

**Table 1:** Factor loads, Average Variance Extracted (AVE*) and reliability values. Brazil, 2014-2015

Variables	Factor load	AVE*	Composite reliability	R^2†^	Cronbach’s alpha
Autonomy	0.59 - 0.80	0.56	0.86		0.80
Control over the environment	0.58 - 0.82	0.56	0.86		0.80
Nursing-physician relationships	0.85 - 0.93	0.81	0.93		0.88
Job satisfaction	0.75 - 0.84	0.62	0.89	0.43	0.85
Safety climate	0.51 - 0.79	0.50	0.85	0.42	0.80
Emotional exhaustion	0.62 - 0.90	0.61	0.93		0.92
Depersonalization	0.55 - 0.79	0.52	0.81		0.68
Decreased personal accomplishment	0.67 - 0.81	0.50	0.87		0.83

*AVE - Average Variance Extracted; †R^2^ - Pearson’s coefficient of determination or explained variance

Regarding discriminant validity, the correlation coefficients values and square root values of the AVE for each of the constructs are presented in Table 2. At this stage of the analysis, it was identified that the factorial load of item 7 of the subscale control over the environment (“The nursing manager is a good manager and leader”) was less than 0.50 and therefore excluded to ensure discriminant validity as recommended in the model analysis.

**Table 2:** Correlation coefficients and mean extracted variance (AVE*). Brazil, 2014-2015

	Variables	1	2	3	4	5	6	7	8
1	Autonomy	0.75^†^							
2	Control over the environment	0.70	0.75^†^						
3	Depersonalization	0.31	0.29	0.72^†^					
4	Emotional exhaustion	0.55	0.55	0.49	0.78^†^				
5	Decreased personal accomplishment	0.50	0.45	0.52	0.63	0.71^†^			
6	Nursing-physician relationships	0.66	0.55	0.20	0.40	0.35	0.90^†^		
7	Safety climate	-0.64	-0.58	-0.32	-0.44	-0.46	-0.45	0.71^†^	
8	Job satisfaction	-0.63	-0.55	-0.32	-0.61	-0.58	-0.51	0.60	0.79^†^

*AVE- Average Variance Extracted; †Square root mean values of the Average Variance Extracted (AVE).

**Figure 1:**
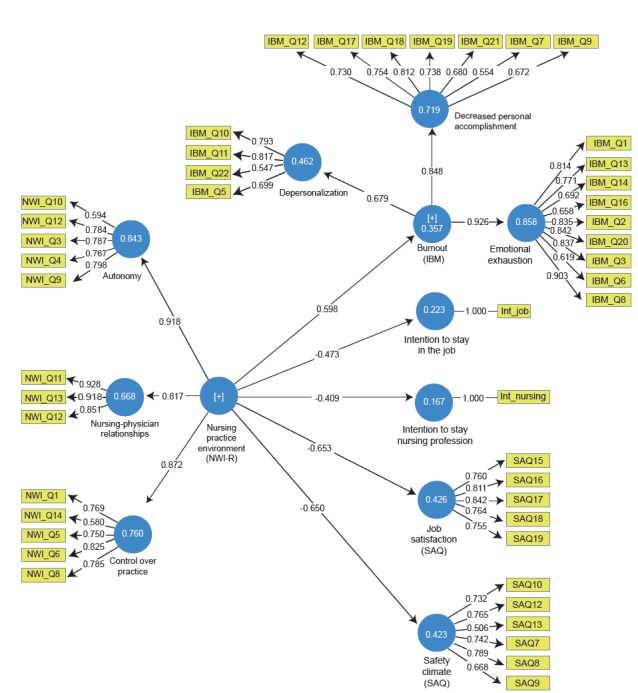
Final structural model

The convergent and discriminant validity of the model was ensured after exclusion of the five items of the model, and the structural model and goodness of fit of the theoretical model were then evaluated (Table 3). The path coefficients values and the significance of the relationships between the constructs are presented in Figure 1 and Table 4.

**Table 3:** Goodness of fit of the final model. Brazil, 2014-2015

Constructs/latent variables	R^2^*	Q^2†^	f^2‡^
Nursing practice environment			0.38
Autonomy		0.47	0.34
Control over the environment		0.42	0.33
Nursing-physician relationships		0.54	0.60
Job satisfaction	0.43	0.25	0.42
Safety climate	0.42	0.20	0.30
Burnout	0.36	0.14	0.35
Emotional exhaustion		0.52	0.51
Depersonalization		0.23	0.21
Decreased personal accomplishment		0.35	0.33
Intention to stay in the current job	0.22	0.22	
Intention to stay in the nursing profession	0.17	0.16	

*R^2^ - explained variance; †Q^2^ - predictive validity or Stone-Geisser indicator; ‡f^2^ - effect size or Cohen indicator

**Table 4:** Evaluation of hypotheses under study. Brazil, 2014-2015

Hypotheses	Path analysis	Path coefficients (Г)	Confidence interval (95%)	Conclusion
H1	Environment → Burnout	-0.60*	-0.533; -0.657	Confirmed
H2	Environment → Intention to stay in the job	0.47*	0.323; 0.487	Confirmed
H3	Environment → Intention to stay in the nursing profession	0.41*	0.394; 0.544	Confirmed
H4	Environment → Job satisfaction	0.65*	0.596; 0.703	Confirmed
H5	Environment → Safety climate	0.65*	0.602; 0.702	Confirmed

*p < 0.0001

## Discussion

Although nurses have a positive perception about the practice environment, demonstrated by the autonomy and good relations between physicians and nurses, they had a negative evaluation of safety climate, were dissatisfied with job, and presented a moderate level of burnout.

The theoretical model required small adjustments to demonstrate adequate reliability and convergent and discriminant validity. The Cronbach’s alpha coefficients and composite reliability values were adequate for all latent variables of the model, noting that composite reliability indices were higher than the Cronbach’s alpha values, which was expected since it does not depend on the number of items of the constructs analyzed^10^.

In the structural model analysis, all relationships between constructs were significant and a strong relationship was identified between the perception of the practice environment and the following variables: job satisfaction, safety climate and burnout. There was also a moderate relationship between the perception of the environment and the intention to stay in the current job and in the nursing profession.

All hypotheses were confirmed using the proposed model test and all constructs showed to be important to the model, as they presented size effect values from moderate to large, as well as adequate predictive validity values, indicating the usefulness of these constructs for the final adjustment.

The constructs of emotional exhaustion and job satisfaction not only had a large explanatory effect but were also very useful in the proposed model. This finding was in line with a research associating the positive characteristics of the practice environment with job satisfaction^2^ ^-^ ^5^ ^,^ ^9^. Other studies have highlighted the contribution of the evaluation of the practice environment and job satisfaction to reduce the intention of nurses to leave their work^13^ ^-^ ^14^. Emotional exhaustion was highlighted as one of the primary dimensions of burnout^15^, since it refers to the feelings of overload and exhaustion of the professionals’ physical and emotional resources.

Regarding the influence of the environment on the perception of safety climate by nurses, the findings were congruent with recent studies^1^ ^,^ ^8^ ^,^ ^15^.

The adjusted model presented high values of variance explained for the job satisfaction (R^2^ = 0.43), safety climate (R^2^ = 0.42) and burnout (R^2^ = 0.36) constructs. The intention to stay in the job was explained in 22% (R^2^ = 0.22) and to stay in the profession, in 17% (R^2^ = 0.17). Perception of the practice environment had a great effect on the explanation of job satisfaction, safety climate and burnout. As for the intention to stay in the current job and in nursing, the variance values explained for the model were of medium magnitude.

The findings are in line with a previous longitudinal study conducted in the United States in which researchers concluded that improvements in the professional practice environment were strongly associated with decreased burnout, reduced intention to leave the job and job dissatisfaction^4^. Other studies indicate that nurses’ positive perception of the practice environment has resulted in increased job satisfaction, lower burnout levels and lower intention to leave the current job and profession^3^ ^,^ ^5^.

The proposed theoretical model showed that the practice environment explained 82% of the variables of work outcomes, such as: nurses’ satisfaction, intention to stay in employment and in the occupation, 42% of the safety climate and 36% of burnout. These values correspond to the explained variance.

This result means that providing an environment where nurses have autonomy, control over the environment and good relationships at work can result in improvements in perception of safety climate and job satisfaction of around 65%, as well as improvements in the intention to stay in the job (up to 47%) and in the nursing profession (41%), as well as reduction of burnout levels (60%). A study that also analyzed the relationship of the practice environment with results at work, considering as mediating variables the workload and burnout, showed that the environment explained 60% of the results at work^13^.

Among the potentialities and limitations of this study, the findings of the relationships between the constructs analyzed by the theoretical model represent only a small part of this complex reality to be interpreted and measured. Although using a study design of limited scope, the present research evidenced that the nurses’ positive perception about the practice environment has a strong impact on the positive perception of safety climate in health institutions. These findings reinforce the hypothesis that organizational behaviors are important in promoting safety climate in health institutions^16^.

One of the limitations implied in working with a larger number of nurses in the sample, as well as the calculation of the response rate in this study, is due to the fact that the participants’ electronic addresses may not be updated. It is also worth mentioning that the researchers did not have access to the data of the participants, for this information is exclusive of the COREN-SP.

We recommend further studies to compare the perception of the nursing practice environment among nurses who provide care and take on management activities so as to enable the identification of specific groups to facilitate the focus for the creation of strategies of management of human resources in nursing.

## Conclusion

All the hypotheses of the theoretical model were confirmed. The model showed that the positive perception of the nursing practice environment exerts a strong impact mainly in the increase of job satisfaction, in the positive perception of safety climate and in reduced levels of burnout.

The importance of evaluating and promoting an environment conducive to nursing practice is reiterated, since strategic actions can positively impact both the results for the professionals and the perception regarding safety climate in care institutions, and the retention of these professionals in their functions.
